# The Effectiveness of a Multidisciplinary Exercise Program in Managing Work-Related Musculoskeletal Symptoms for Low-Skilled Workers in the Low-Income Community: A Pre-Post-Follow-Up Study

**DOI:** 10.3390/ijerph16091548

**Published:** 2019-05-02

**Authors:** Kin Cheung, Mimi M. Y. Tse, Chi Kan Wong, Kwan Wai Mui, Siu Kan Lee, Ka Yan Ma, Keith T. S. Tung, Echo Ping Woi Lau

**Affiliations:** 1 School of Nursing, The Hong Kong Polytechnic University, Hong Kong, China; mimi.tse@polyu.edu.hk (M.M.Y.T.); eunis.ma@polyu.edu.hk (K.Y.M.); keithtung144@gmail.com (K.T.S.T.); echo.lau@polyu.edu.hk (E.P.W.L.); 2 Caritas Community Development Service, Hong Kong, China; wongchikan64@gmail.com (C.K.W.); muiterence@caritassws.org.hk (K.W.M.); leesiukan@caritassws.org.hk (S.K.L.)

**Keywords:** public health, health promotion, occupational health, social class, health inequalities

## Abstract

Studies on work-related musculoskeletal symptoms (WRMSs) have been conducted mainly on different types of workforce but not many on low-skilled workers. The purpose of this study was to evaluate the effectiveness of a multidisciplinary exercise program in decreasing the number of body parts with WRMSs for low-skilled workers. This study used a repeated-measures, single-group design. One hundred and five (105) workers participated in eight weekly 90-min sessions (including 45-min workshops and 45-min exercises) in low-income community settings. The exercise program involved a 21-movement stretching exercise and a 10-movement muscle-strengthening exercise. Questionnaire and health-assessment data were collected at the baseline (N = 105) and immediately after the 8-week program (n = 86). The average age of the 105 participants was 50.5 ± 8.7 years (ranging from 31 to 67). Over 80% (n = 87) of them were female, 68.6% (n = 72) were married, and 68.6% (n = 72) had completed secondary school. They reported an average of three body parts with WRMSs at baseline (T0). By the end of the eight weeks (T1), the participants had reduced the number of WRMS-affected body parts, job stress, and incidences of working through pain, and had improved spine flexibility and handgrip strength. The factors significantly affecting the reduction in the number of body parts with WRMSs were change in the workstyle of working through pain, and self-rated health status. Our study has demonstrated that a community-based multidisciplinary program can reduce the number of body parts affected by WRMSs in low-skilled workers in low-income communities.

## 1. Introduction

Worldwide, work-related musculoskeletal symptoms (WRMSs) are a major public health issue, with musculoskeletal conditions contributing the greatest proportion of lost work productivity [1]. Workers with WRMSs may experience pain, numbness, stiffness, and aching in various body parts [2]. Of all working adults, grassroots working-class workers are the most vulnerable to WRMSs because the nature of their work mostly exposes them to the identified risk factors [3].

Evidence has shown multidimensional programs, including exercise, can prevent/manage WRMSs in different contexts: office workers in Portugal [4]; bus drivers in South Korea [5]; workers requiring prolonged standing in Portugal [6]; and underprivileged migrant workers in a community setting in Korea [7]. A review of 61 studies of the effectiveness of workplace intervention for WRMSs showed that exercise programs, such as stretching and resistance-training, have demonstrated positive effects with moderate-to-strong levels of evidence [8]. Although nearly 60% of the WRMS intervention studies were conducted in office-based workplaces, the review indicated that it would be possible to implement the interventions in non-office settings.

Although the workplace has been recommended as a place for promoting physical activity, this concept is not yet accepted widely [9], particularly in small or medium-sized enterprises. Low-skilled workers might not be a beneficiary group because their workplaces most likely do not offer WRMSs prevention/management programs. In addition to their low education levels, this group might have limited access to WRMSs prevention/management knowledge. Community-based WRMSs prevention/management programs could be an innovative way to target low-skilled workers. Despite limited relevant studies having been conducted, one community-based study with Korean–Chinese migrant workers has shown promising results [7]. These Korean–Chinese migrant workers (of whom 90% were domestic or restaurant workers) rarely received any interventions, such as stretching exercises, which are commonly provided to native Koreans in their workplaces. The results of this randomized control trial study were encouraging, and suggested that community-based stretching exercise programs are feasible and effective in helping low-skilled working-class workers to prevent/manage WRMSs [7].

The aim of this study was to evaluate the effectiveness of a multidisciplinary exercise program in managing WRMSs for low-skilled workers in low-income community settings. The hypotheses were that the workers would reduce the number of their body parts with WRMSs (primary outcomes) and improve their workstyles, self-rated health status, self-rated job stress, self-efficacy, social support, mental health, handgrip strength, and spine flexibility (secondary outcomes) between baseline (T0) and immediate (T1) follow-up measures after the program.

## 2. Methods

### 2.1. Study Design, Setting, and Participants

This was a repeated-measures, single-group design study. The participants were recruited from low-income communities through seven Caritas Community Development Service (CCDS) [10] centers from all three regions of Hong Kong. The inclusion criteria were low-skilled workers aged ≥ 18 working full-time or part-time, with at least one body part that had been affected by WRMSs for at least one month. A “low-skilled” job was defined as work not requiring a high level of skill or without formal qualification requirements [11]. This type of job usually plays a supporting role in an organization [12]. Some of the typical low-skilled jobs are cleaning, laboring, general office work, equipment operation, and helping in restaurants [12,13,14]. The exclusion criteria were engagement in WRMS prevention programs in the workplace at the time of the study, undergoing medical treatment, or doing stretching exercises more than three times a day, five days per week [7].

### 2.2. Multidisciplinary Exercise Program

Ethical approval was obtained from The Hong Kong Polytechnic University (HSEARS20170815005). The program involved eight weekly 90-min sessions, with a group size of 13–22 participants. Each session consisted of a 45-min workshop and then a total of 45 min of stretching (10.5–15 min) and muscle strengthening (5–10 min) exercises, followed by a question-and-answer period (about 20 min).

The eight WRMS workshop topics, conducted by nurses, physiotherapists, traditional Chinese medicine (TCM) doctor, and social workers, included: (1) Exercise; (2) Health and work; (3) Manual handling; (4) Non-pharmaceutical pain management; (5) TCM; (6) Mental health; (7) Laws and compensation; and (8) Community resources. The teaching material was validated by a panel of seven experts representing the fields of nursing, occupational health, TCM, physiotherapy, and social workers. The content validity of the teaching material was 1.00.

The exercise regime was based on exercises suggested by occupational safety and health authorities [15,16] and validated by four physiotherapists and three nurses. The 21-movement stretching exercise involved neck (6 movements), shoulders (1), upper extremities (2), wrists (4), back (6), and lower extremities (2). The 10-movement muscle strengthening exercise involved neck (6 movements), upper extremities (2) and lower extremities (2). Each movement took 10 seconds and was repeated three times.

The data were collected from a questionnaire (30 min) and health assessments (30 min) conducted at the baseline (T0) and immediately after the program completion (T1). To ensure the consistency of the data-collection procedure, the personnel responsible for collecting the data in each center were trained for two hours by a member of the research team, and their return demonstrations achieved satisfactory results. The same models of equipment were used for the health assessments in each center, to ensure consistency.

### 2.3. Measurements

The questionnaire was developed based on previous studies and a literature review [17,18,19,20,21,22,23,24,25]; it was validated by the same panel as the teaching material. The content validity index was 0.99. Some parts of the questionnaire had been tested previously by the investigators or in other local studies. Below is a description of each section:

1. Personal information included age, gender, education level, and job-related information. Self-rated health status was measured with a 4-point Likert scale (from 0 = very poor to 3 = very good). In addition, self-rated job stress was collected using a 5-point Likert scale (from 0 = none to 4 = very stressful).
2. Self-reported musculoskeletal symptoms were assessed by the general Nordic Musculoskeletal Questionnaire (NMQ) about pain, aches or discomfort in different body parts [2]. The NMQ has been used to evaluate the effect of intervention programs in reducing musculoskeletal symptoms [17,18], and it has also been used by the research team with local nursing personnel [18,19,20].
3. The WRMS knowledge scale, developed by the research team [20], measured knowledge about ergonomic principles, and manual handling. The scale’s validity and reliability had been established prior to the study through trialing with local workers [20]. The knowledge scores were tabulated for data analysis by summing the correct responses. Higher scores represented better knowledge. The Cronbach’s alpha was 0.60.
4. Workstyle was assessed by using the 24-item Chinese Workstyle Short Form (C-WSF) for WRMSs with a 5-point Likert scale (from 0 = almost never to 4 = almost always) [20]. Workstyle is generally defined as an interactive pattern of a particular worker’s behavioral, physiological and cognitive responses to their work demands with both ergonomic and psychological risk factors in work environments [21]. The sums of the subscales were used, with a high score indicating a high frequency of adverse workstyle practices. The internal consistency coefficients for the four subscales were: 0.83 for working through pain (e.g., I feel aching while at work), 0.91 for social reactivity (e.g., I cannot interrupt my work because my other team members will be unhappy with me), 0.88 for demands at work (e.g., I have too many deadlines and I can never finish my work) and 0.52 for breaks (e.g., while at work I occasionally stop working to take a break) [20]. The Cronbach’s alpha for the overall C-WSF was 0.91.
5. Self-efficacy for exercise was assessed by two subscales: (a) the 12-item self-efficacy for exercise behaviors [22], and (b) the 5-item self-efficacy scale [23] measuring an individual’s confidence for exercise behavior change. Both used 5-Likert scales ranging from “not at all confident” to “extremely confident”. The sums of subscales were yielded, with higher scores representing higher levels of self-efficacy for exercise. The Cronbach’s alphas for the two subscales were 0.93 and 0.84, respectively.
6. Social support for exercises was assessed by using the 10-item Chinese Social Support and Exercise (SSE) questionnaire with a 5-point Likert scale (0 = “never” to 4 = “very often”) [24]. A score for the items in each scale was summated; the higher the score, the greater the level of support. The Cronbach’s alphas for family and friends were 0.91 and 0.95, respectively.
7. Depression and anxiety status were assessed to indicate each participant’s mental health. Depression was assessed by using the 20-item Center for Epidemiologic Studies Depression Scale (CES-D) with a 4-point Likert scale (0 = rarely or none of the time, to 3 = applied to me very much or most of the time). This measured the frequency of common depressive symptoms over the preceding week. The Chinese version of CES-D had been tested previously with local Chinese patients and good reliability was established (α = 0.85) [25]. The scores of all items were summed for the data analysis. The cut-off point for possible depression was ≥ 16. Anxiety was assessed by the criteria from DSM-V. This measured the common anxiety symptoms over the six months [26]. The Cronbach’s alphas for depression and anxiety were 0.90 and 0.78, respectively.
8. Six extra items were added to the T1 questionnaire to evaluate the appropriateness of the program’s content level, usefulness, teaching pace and session times, the extent to which the program met their expectations, and their overall satisfaction. The items used a 4-point Likert scale of 1 (“poor or useless”) to 4 (“very good or very useful”).

Spine flexibility was assessed using the sit-and-reach method. The subjects were instructed to sit on the floor with both legs straight and their soles touching the sit-and-reach box. They were then asked to bend their backs, with both palms facing down and fingertips straight ahead, to push the scale on the top of the box. The distance pushed was measured in centimeters. Other health assessments, such as body mass index (BMI), hip-waist ratio (HWR), blood pressure (BP), blood glucose (BG), cholesterol (using a glucometer), and handgrip strength, were also measured.

### 2.4. Data Analyses

All the analyses were computed by using the Statistical Package for Social Sciences (SPSS, version 24.0). To examine what factors influenced the number of body parts with WRMSs, which was treated as a continuous variable, at the baseline, non-parametric Spearman correlation analyses were used. A series of linear regression analyses with all variables entered at once into the regression model were employed. Factors found to be correlated significantly in the non-parametric test were entered into the univariate regression analyses, and a multivariate regression model was used to identify the contributing factors. The Wilcoxon Signed Ranks tests were adopted to observe any significant changes in the number of body parts with WRMSs and associated factors over the eight-week period. Non-parametric Spearman correlation analyses were then conducted to test whether there was any association between the measured factors and the changes in the number of body parts with WRMSs. For all significant correlated factors, mutually adjusted multiple linear regression analyses were employed, with age and gender included in the model as covariates, to confirm what factors truly contributed to the reduction in the number of body parts with WRMSs. Statistical significance was determined by two tailed tests, with a *p*-value of < 0.05.

## 3. Results

### 3.1. Characteristics of Participants at Baseline (T0)

The average age of the 105 participants was 50.5 ± 8.7 years (ranging from 31 to 67). Over 80% (n = 87) of them were female, 68.6% (n = 72) were married, and 68.6% (n = 72) had completed secondary school. Regarding their employment status, 30.5% (n = 32) were part-time employees. They reported having experienced WRMSs in an average of three body parts at T0. In addition, female workers with depression or anxiety had more body parts with WRMSs (*p* < 0.05). No significant differences were observed among other demographic characteristics (Table 1).

### 3.2. Factors Associated with Body Parts with WRMSs at Baseline (T0)

Table 2 shows that the number of body parts with WRMSs was significantly correlated with the workstyle of working through pain, social reactivity, the total score for workstyle, self-rated health status, and depressive symptoms.

Under univariate regression analyses, gender, the workstyle of working through pain, social reactivity, depressive symptoms, anxiety and self-rated health status were all found to be significantly associated with the number of body parts with WRMSs. Multivariate regression analysis indicated that gender and the workstyle of working through pain remained as significant factors. Workstyle seems to have been the most influential factor in affecting the number of body parts with WRMSs (Table 3).

### 3.3. Factors Associated with the Change before and after Intervention (T1-T0)

Immediately after the intervention (T1), 86 (81.9%) participants took part in the follow-up data collection. However, there were no significant differences found in the reported number of body parts with WRMSs between the participants who joined T1 and those who dropped out. Table 4 shows that the number of body parts with WRMSs, sit-and-reach distance, handgrip strength, workstyle of working through pain, and self-rated job stress had changed significantly over time. The number of body parts with WRMSs was reduced by a mean value of 1.05. The workstyle of working through pain and self-rated job stress was also reduced significantly from T0 to T1 (−1.70 and −0.29, respectively). Also, both the sit-and-reach distance and the right-hand grip strength were enhanced by mean values of 1.95 cm and 0.40 kg, respectively, at T1.

Under the univariate model, it was demonstrated that gender and change in the workstyle of working through pain and right-hand grip strength were significantly associated with the change in the number of body parts with WRMSs. After adjusting for potential confounders, changes in the workstyle of working through pain and self-rated health status were the significant factors influencing the reduction in the number of body parts with WRMSs (Table 5).

### 3.4. Evaluation of the Program

With a response rate of 93.02% (n = 80), the participants rated all aspects of the program positively, with means ranging from 3.33 to 3.44 out of 4.00.

## 4. Discussion

Our study is one of the first to develop a WRMS-prevention multidisciplinary program with stretching and muscle-strengthening exercises for low-skilled workers in a low-income community setting. Our results have provided promising evidence of the 2-month program’s effectiveness in reducing the number of body parts with WRMSs, job stress, working through pain, improved spine flexibility, and handgrip strength. The factors significantly affecting the reduction in the number of body parts with WRMSs were the change in the workstyle of working through pain, and self-rated health status. In addition, our analyses demonstrated a medium effect size (0.44) with a good statistical power of 0.98.

Our results have also strengthened the evidence that the community could be the setting for WRMSs prevention programs, apart from workplaces. Consistent with Lee et al.’s study [7] in a Korean community, our program not only improved workers’ flexibility but also reduced WRMSs and other outcomes. The success of our program could be due to its partnership design, multidisciplinary and multidimensional approaches, and appropriate length (right dose). Studies have shown the ineffectiveness of single interventions (such as ergonomics training or stress management); rather, multidimensional interventions are recommended to tackle the multifactorial nature of WRMSs [4,5,6,8]. The element of TCM also addressed the cultural needs of the Chinese participants. Furthermore, our exercise duration (15.5–25 min) was longer than that of the Korean study (6 min) [7]. Thus, the community center could serve as a vehicle to reach low-skilled, disadvantaged workers, to allow them to access the WRMSs prevention programs, and to bring them together synergistically [27].

The Workstyle model [21] and the concept of presenteeism (continuing to work despite not feeling well) [28] could be used to explain our results further. Our program might have been able to improve low-skilled workers’ musculoskeletal health literacy. For instance, they became more aware that WRMSs are not a necessary consequence of their work, that they are human beings not instruments or machines in the workplace, and that they should take an active role in improving their musculoskeletal health. They might have changed from passivity to becoming active in improving their own WRMSs. Using the Workstyle model, adverse workstyles have been identified as factors associated with WRMSs in office workers [21,29,30] and nursing assistants in nursing homes [31]. Also, working through pain [20,32], self-rated health [20], and self-rated mental health [32] have been found to be associated with WRMSs. Workers with WRMSs tended to have higher presenteeism (such as working through pain) and vice versa [33]. Furthermore, more WRMSs and higher presenteeism have been found to be related to low-skilled workers, low education, low job resources, and longer working hours, and to be more common in female workers [33]. In addition, high presenteeism has been associated with low self-rated health [28,33], and workers with low self-rated health tended to have more WRMSs [33]. Moreover, a population-based study (N = 1615) found that workers with long working hours, working without contracts, and being highly dependent on their wages to contribute to the total household income were associated with higher presenteeism [34]. Reasons given for presenteeism included not wanting to burden colleagues, not being able to afford to be absent for economic reasons, and worrying about being laid off [34]. The factors associated with presenteeism identified in the literature match with the general characteristics of low-skilled workers. Based on the Workstyle model, working with pain is a behavior resulting from limited job resources, financial need to support families, and fear of losing the job (cognitive reasoning and psychological considerations) [21,29]. Additionally, the East Asian hierarchical work structure weakens employees’ power to question their seniors or those at management level [32]. Chinese culture also emphasizes self-discipline and the individual’s responsibility to others and to society [35]. Because low-skilled workers are at the bottom of the organizational structure [36], they have low bargaining power and are expected to adhere to their job duties and be loyal to the organization. The promising results of our program reflect that the elements of the workshops might have changed the participants’ cognitive and psychological appraisals (part of the Workstyle model), with an understanding that presenteeism (working while suffering from pain) could aggravate their WRMSs further.

This study was limited by its single-group pre-and-post study design, with the majority of our participants being female. This might temper the generalizability of our findings. However, we attempted to enhance the representativeness of the study population by including seven different community centers as recruitment sites. Nevertheless, this study provides preliminary evidence for the effectiveness of the program and gives rise to further investigations. A clustered random controlled trial with a robust design, such as a larger sample size with well-matched interventions and a control group, is needed to build up generalizable and representative evidence for the effectiveness of this multidisciplinary exercise intervention program.

There are implications for practice: The findings of our study can help to inform policymakers, employers, and occupational health and safety stakeholders to pay attention to WRMSs, particularly among low-skilled workers. Indeed, the promising results of our study suggest that more resources from policymakers should be allocated to community centers (e.g., non-governmental organizations) to conduct WRMS prevention and management programs. Further studies can explore the concept of the settings approach to improve low-skilled workers’ musculoskeletal health literacy. The four core principles of the settings approach, namely, community participation, partnership, empowerment and equity [37], can be used as a framework to guide community-based interventions. Empowerment refers to “the process by which people gain control over the factors and decisions that shape their lives” [38]. Through community participation and partnership with different occupational health and safety stakeholders and employers, community-based programs can empower workers to take control of their own musculoskeletal health. Self-help groups, or even musculoskeletal health ambassadors, could be established in community centers. Those groups or ambassadors could provide further social support to their peers using social media (such as WhatsApp, Line, or WeChat). Addressing musculoskeletal health successfully in low-skilled workers could give them more equitable access to health promotion and to lobby for more resources allocated to communities. The equity approach would enable every low-skilled worker to have the opportunity to access musculoskeletal health and services.

## 5. Conclusions

Community could be the alternative setting for WRMS prevention programs for low-skilled workers. Our study has demonstrated that a community-based multidisciplinary program can reduce the number of body parts affected by WRMSs in low-skilled workers in low-income communities. Further studies should be conducted to test the program. Healthcare professions and policymakers should explore the concept of the settings approach to allocate resources to community centers in order to empower low-skilled workers to lobby their right for musculoskeletal health.

## Figures and Tables

**Table 1:** Characteristics of participants, and the association of factors and number of body parts with WRMSs at baseline (T0).

Factors			Number of Painful Body Parts	Comparison #
	N	%	Median	*p*
TOTAL	105	100.0	3	-
Gender				0.013 *
Male	18	17.1	2	
Female	87	82.9	4	
Marital status				0.239
Single	12	11.4	3	
Married	72	68.6	3	
Divorced	14	13.3	4.5	
Widowed	7	6.7	4	
Education level				0.627
Primary school	26	24.8	3	
Secondary school	72	68.6	3	
Tertiary institute	5	4.8	4	
University or above	2	1.9	2	
Smoking				0.189
Yes	8	7.6	2.5	
No	97	92.4	3.0	
Drinking				0.812
Yes	3	2.9	4	
No	102	97.1	3	
Stretching				0.948
Yes (≤3 times/day)	48	45.7	3	
No	57	54.3	3	
Need to work overtime				0.246
Yes	18	17.1	4	
No	83	79.0	3	
Job type				0.117
Full time	73	69.5	3	
Part time	32	30.5	4	
Job nature				0.979
Mild labour-intensive job (craft and related worker, sales and service worker, clerical support worker)	52	49.5	3.5	
Labour-intensive job (machine operator, elementary worker, others)	53	50.5	3	
Depression (dichotomous)				0.006 **
Yes	31	29.5	4	
No	73	69.5	3	
Anxiety (dichotomous)				0.012 *
Yes	17	16.2	5	
No	88	83.8	3	

# Mann–Whitney test or Kruskal–Wallis test, whichever appropriate. *: *p* < 0.05, **: *p* < 0.01.

**Table 2:** Correlation table with the number of body parts with WRMSs at baseline (T0).

Variables	*r*	*p*	
Work-related musculoskeletal health knowledge	0.017	0.867	
Work style—Work through pain	0.457	<0.001	***
Work style—Social reactivity	0.298	0.002	**
Work style—Demands at work	0.177	0.074	
Work style—Break	−0.154	0.117	
Work style—Total score	0.295	0.002	**
Self-efficacy for exercise	−0.134	0.174	
Social support from family	−0.036	0.717	
Social support from friends	0.043	0.665	
T0_Sbp_1_mmHg	−0.105	0.286	
T0_Dbp_1_mmHg	0.004	0.964	
T0_pulse_1_bpm	0.116	0.237	
BMI	0.002	0.985	
Wrist-to-hip ratio	0.083	0.400	
Body fat percentage	−0.008	0.936	
Blood sugar level	−0.009	0.926	
Cholesterol level	0.072	0.489	
Sit and reach	0.003	0.977	
Hand grip strength (right)	−0.076	0.443	
Hand grip strength (left)	−0.025	0.799	
Age	−0.077	0.433	
Self-rated health status	−0.346	<0.001	***
Depressive symptoms (score)	0.254	0.009	**
Self-rated job stress	0.182	0.065	

**: *p* < 0.01, ***: *p* < 0.001.

**Table 3:** Univariate and multivariate regression model studying factors affecting the number of body parts with WRMSs at baseline (T0).

Factors Affecting the Number of Painful Body Parts	Univariate			Multivariate #		
	B	*p*		B	*p*	
Gender	1.467	0.014	*	1.538	0.005	**
Working style						
Work through pain	0.199	0.000	***	0.146	0.004	**
Social reactivity	0.151	0.003	**	0.015	0.793	
Depressive symptoms (score)	0.061	0.005	**	0.009	0.714	
Anxiety (dichotomous)	1.305	0.039	*	0.628	0.331	
Self-rated health status	−1.531	0.000	***	−0.633	0.145	

# Mutually adjusted for each other. *: *p* < 0.05, **: *p* < 0.01, ***: *p* < 0.001.

**Table 4:** Changes in the number of body parts with WRMSs and associated factors over time (T1−T0).

	Baseline—Mean	T1—Mean	Changes (T1−T0)	*p #*	
Number of body parts with WRMSs	3.82	2.77	−1.05	<0.001	***
Sit and reach	25.60	27.55	1.95	<0.001	***
Hand grip (left) (kg)	21.45	21.33	−0.12	0.005	**
Hand grip (right) (kg)	22.75	23.15	0.40	0.001	**
Depression symptoms	12.60	12.18	−0.42	0.578	
Number of workers with anxiety	17	17	0	-	
Self-efficacy for exercise	1.17	1.25	0.08	0.591	
Social support from family	1.02	1.09	0.07	0.237	
Social support from friends	0.89	1.00	0.11	0.880	
Self-rated job stress	1.62	1.33	−0.29	0.027	*
Workstyle—total score	35.64	33.50	−2.14	0.706	
Work through pain	11.93	10.23	−1.70	0.005	**
Social reactivity	5.15	4.84	−0.31	0.794	
Demands at work	16.02	15.74	−0.28	0.538	
Break	2.59	2.68	0.09	0.187	
Self-rated health status	1.60	1.72	0.12	0.197	

# Wilcoxon signed-ranks test. *: *p* < 0.05, **: *p* < 0.01, ***: *p* < 0.001.

**Table 5:** Univariate and multivariate regression model studying factors affecting the change in the number of body parts with WRMSs over time (T1−T0).

Factors Affecting the Number of Body Parts with WRMSs +	Univariate			Multivariate #		
	B	*p*		B	*p*	
Age	0.460	0.097		0.059	0.031	*
Gender	−1.514	0.038	*	−1.618	0.029	*
Working style						
Work through pain	0.209	<0.001	**	0.265	<0.001	***
Social reactivity	−0.024	0.757		−0.092	0.379	
Sit and reach	−0.029	0.641		−0.091	0.104	
Handgrip strength (left)	−0.051	0.315		−0.116	0.108	
Handgrip strength (right)	−0.118	0.013	*	−0.044	0.428	
Depressive symptoms (score)	0.009	0.814		0.011	0.767	
Anxiety (dichotomous)	0.063	0.919		−0.542	0.387	
Self-rated job stress	0.440	0.083		0.346	0.221	
Self-rated health status	−0.094	0.863		1.161	0.031	*

+ All factors refer to the changes over time (T1−T0) in Table 4, except gender, depressive symptoms, self-rated health status, and anxiety (found significant in T0 in Table 1, Table 2 and Table 3) and age. # Mutually adjusted for each other. *: *p* < 0.05, **: *p* < 0.01, ***: *p* < 0.001.

## References

[1] World Health Organization Musculoskeletal Conditions 2018. http://www.who.int/news-room/fact-sheets/detail/musculoskeletal-conditions.

[2] Kuorinka I., Jonsson B., Kilbom A., Vinterberg H., Biering-Sørensen F., Andersson G., Jørgensen K. (1987). Standardised Nordic questionnaires for the analysis of musculoskeletal symptoms. Appl. Ergon..

[3] Summers K., Jinnett K., Bevan S., The Center for Workforced Health and Performance (2015). Musculoskeletal Disorders, Workforce Health and Productivity in the United States.

[4] Machado-Matos M., Arezes P.M. (2016). Impact of a workplace exercise program on neck and shoulder segments in office workers. Dyna.

[5] Lee J.H., Gak H.B. (2014). Effects of self stretching on pain and musculoskeletal symptom of bus drivers. J. Phys. Ther. Sci..

[6] Moreira-Silva I., Santos R., Abreu S., Mota J. (2014). The effect of a physical activity program on decreasing physical disability indicated by musculoskeletal pain and related symptoms among workers: A pilot study. Int. J. Occup. Saf. Ergon..

[7] Lee H., Chae D., Wilbur J., Miller A., Lee K., Jin H. (2014). Effects of a 12 week self-managed stretching program among Korean–Chinese female migrant workers in Korea: A randomized trial. Jpn. J. Nurs. Sci..

[8] Van Eerd D., Munhall C., Irvin E., Rempel D., Brewer S., Van Der Beek A.J., Dennerlein J.T., Tullar J., Skivington K., Pinion C. (2016). Effectiveness of workplace interventions in the prevention of upper extremity musculoskeletal disorders and symptoms: An update of the evidence. Occup. Environ. Med..

[9] World Health Organization (2018). The Workplace: A priority Setting for Health Promotion. http://www.who.int/occupational_health/topics/workplace/en/.

[10] Caritas Community Development Service (2018). Caritas Community Development Service. http://cd.caritas.org.hk/02aboutus.htm.

[11] Ruhle S.A., Suess S. (2018). Presentieeism and Low-Skilled Work—A Conceptual Analysis. Academy of Management Proceedings. Acad. Manag. Proc..

[12] Human Resources and Skills Development Canada (2011). National Occupational Classification Matrix 2011. http://www.immigrationcanadaservices.com/wp-content/uploads/2016/11/Matrix-1.pdf.

[13] Immigration Canada Services (2017). Job Opportunities for Low Skilled Workers in Canada. http://www.immigrationcanadaservices.com/job-opportunity-for-low-skilled-workers-in-canada/.

[14] Migration Advisory Committee (2014). Migrants in Low-Skilled Work: The Growth of EU and Non-EU Labour in Low-Skilled Jobs and its Impact on the UK. https://www.gov.uk/government/organisations/migration-advisory-committee.

[15] Occupational Safety and Health Branch Labour Department (2007). More Exercise Smart Work II. https://www.labour.gov.hk/eng/public/oh/MESW.pdf.

[16] Occupational Safety & Health Council (2010). Stretching Exercise to Prevent Occupational Musculoskeletal Disorders. http://www.oshc.org.hk/oshc_data/files/leaflets/2016/CL1319C.pdf.

[17] Svensson A.L., Strøyer J., Ebbehøj N.E., Schultz-Larsen K., Marott J.L., Mortensen O.S., Suadicani P. (2009). Multidimensional intervention and sickness absence in assistant nursing students. Occup. Med..

[18] Szeto G.P., Law K.Y., Lee E., Lau T., Chan S.Y., Law S.W. (2010). Multifaceted ergonomic intervention programme for community nurses: Pilot study. J. Adv. Nurs..

[19] Cheung K. (2010). The incidence of low back problems among nursing students in Hong Kong. J. Clin. Nurs..

[20] Cheung K., Szeto G., Lai G.K., Ching S.S. (2018). Prevalence of and factors associated with work-related musculoskeletal symptoms in nursing assistants working in nursing homes. Int. J. Environ. Res. Public Health.

[21] Feuerstein M. (1996). Workstyle: Definition, Empirical Support, and Implications for Prevention, Evaluation, and Rehabilitation of Occupational Upper-Extremity Disorders (Chapter 11). Beyond Biomechanics: Psychosocial Aspects of Musculoskeletal Disorders in Office Work.

[22] Sallis J.F., Pinski R.B., Grossman R.M., Patterson T.L., Nader P.R. (1988). The development of self-efficacy scales for health-related diet and exercise behaviors. Health Educ. Res..

[23] Marcus B.H., Selby V.C., Niaura R.S., Rossi J.S. (1992). Self-efficacy and the stages of exercise behavior change. Res. Q. Exerc. Sport.

[24] Liang Y., Lau P.W., Huang W.Y., Maddison R., Baranowski T. (2014). Validity and reliability of questionnaires measuring physical activity self-efficacy, enjoyment, social support among Hong Kong Chinese children. Prev. Med. Rep..

[25] Zhang Y., Ting R.Z., Lam M.H., Lam S.P., Yeung R.O., Nan H., Ozaki R., Luk A.O., Kong A.P., Wing Y.K. (2015). Measuring depression with CES-D in Chinese patients with type 2 diabetes: The validity and its comparison to PHQ-9. BMC Psychiatry.

[26] American Psychiatric Association (2013). Desk reference to the diagnostic criteria from DSM-5 (DSM-5). (Taiwanese Society of Psychiatry, Trans).

[27] World Health Organization (2018). Introduction to Healthy Settings. https://www.who.int/healthy_settings/about/en/.

[28] Skagen K., Collins A.M. (2016). The consequences of sickness presenteeism on health and wellbeing over time: A systematic review. Soc. Sci. Med..

[29] Feuerstein M., Huang G.D., Pransky G. (1999). Workstyle and Work—Related Upper Extremity Disorders. Psychosocial Factors in Pain.

[30] Sharan D., Parijat P., Sasidharan A.P., Ranganathan R., Mohandoss M., Jose J. (2011). Workstyle risk factors for work related musculoskeletal symptoms among computer professionals in India. J. Occup. Rehabil..

[31] Cheung K., Ching S.S., Ma K.Y., Szeto G. (2018). Psychometric Evaluation of the Workstyle Short Form among Nursing Assistants with Work-Related Musculoskeletal Symptoms. Int. J. Environ. Res. Public Health.

[32] Maakip I., Keegel T., Oakman J. (2015). Workstyle and musculoskeletal discomfort (MSD): Exploring the influence of work culture in Malaysia. J. Occup. Rehabil..

[33] Choi S., Yi Y., Kim J. (2018). Exposure to adverse social behavior in the workplace and sickness presenteeism among Korean workers: The mediating effects of musculoskeletal disorders. Int. J. Environ. Res. Public Health.

[34] Navarro A., Salas-Nicás S., Moncada S., Llorens C., Molinero-Ruiz E. (2018). Prevalence, associated factors and reasons for sickness presenteeism: A cross-sectional nationally representative study of salaried workers in Spain, 2016. BMJ Open.

[35] Wu K. (2014). Research on Sense of Social Responsibility in China: Looking Back and Looking Forward. Cross-Cult. Commun..

[36] Maxwell N.L. (2006). Low-Skilled Jobs: The Reality Behind the Popular Perceptions.

[37] World Health Organization (2018). Healthy Settings. https://www.who.int/healthy_settings/en/.

[38] World Health Organization (2018). Track 1. Community Empowerment. https://www.who.int/healthpromotion/conferences/7gchp/track1/en/.

